# Non-bacterial Thrombotic Endocarditis as a Rare Manifestation of Early Stage Gastric Cancer

**DOI:** 10.7759/cureus.25213

**Published:** 2022-05-22

**Authors:** Jorge R Fernandes, Ana C Rodrigues, Vera R Bernardino, António Panarra

**Affiliations:** 1 Internal Medicine Department, Curry Cabral Hospital, Central Lisbon University Hospital Centre, Lisbon, PRT

**Keywords:** tricuspid valve endocarditis, gastric cancer, rare condition, thrombophilia, non-bacterial thrombotic endocarditis

## Abstract

Endocarditis is an inflammation of the endocardium and is characterized by the presence of vegetation, which may occur in the context of infectious or non-infectious diseases. Despite the higher rate of infective endocarditis diagnosis, it may also surge in other non-infectious conditions such as cancer or chronic inflammatory syndromes. Cancer defines a hypercoagulable state, and cancer-associated thrombophilia can have a diverse clinical presentation, most commonly venous thromboembolism and rarely non-bacterial thrombotic endocarditis (NBTE). The diagnosis of NBTE is difficult and requires a high level of suspicion. The treatment relies on anticoagulant therapy, control of underlying disease, and valve replacement when applied. Independently of the etiology, without treatment, endocarditis may lead to valve dysfunction and to the worst prognosis. In this paper, we describe a case of a patient with persistent fever and NBTE of the tricuspid valve, disclosing a rare presentation of gastric cancer.

## Introduction

Nonbacterial thrombotic endocarditis (NBTE) is a rare clinical entity in which sterile fibrin deposits develop on heart valves [1]. A triad of immune complex formation, carcinomatosis, and a hypercoagulable state which, independently or altogether, may cause endocardial damage and explain NBTE [2]. Malignancies are strongly associated with a hypercoagulable state, with up to 15% of cancer patients having thrombotic phenomena and up to 50% in autopsy series [2-4]. Adenocarcinoma of the pancreas, lung, and stomach appears to be the most common histological type and cancer locations among NBTE patients [2,5-7]. At the time of NBTE diagnosis, most patients already have metastatic disease, which may explain a higher mortality rate. We report a challenging case of NBTE in a patient with persistent fever in the context of gastric cancer.

## Case presentation

A 79-year-old woman was admitted to the hospital with a one-month history of persistent fever (38.5-39.5ºC) associated with migraines and nocturnal sweating, inflammatory arthralgia of the proximal inter-phalangeal of the third and fourth metacarpal (bilateral) and scapular myalgia without myopathy. She also reported asthenia, anorexia, and weight loss in the previous year (11% of her total body weight). She had already been treated for presumed cystitis with ciprofloxacin and then pyelonephritis with cefuroxime, with no clinical response. Blood and urine cultures were negative at that time. Her clinical background was relevant for well-controlled long-standing hypertension treated with amlodipine, takotsubo cardiomyopathy one year before, and allergy to penicillin. Her family history was negative for cancer or autoimmune diseases. Other epidemiological and infectious risk factors were excluded, including those related to sexually transmittable diseases, drug consumption, or contact with domestic or non-domestic animals.

On admission, she presented with normal vital signs, and the physical exam was unremarkable except for some degree of emaciation. Laboratory evaluation revealed normocytic and normochromic anemia (hemoglobin 10.4 g/dL), mild neutrophilia (8.56 x 10^9^/L), erythrocyte sedimentation rate of 90 mm/h, C-reactive protein of 160.6 mg/L, thyroid-stimulating hormone of 0.06 mUI/mL, with low free T3 and normal free T4. At this point, the differential diagnosis included infectious diseases (including bacterial, viral, fungal, and zoonotic infections), cancer, and autoimmune etiologies (including systemic lupus erythematosus, antiphospholipid syndrome, and rheumatoid arthritis).

Serology for human immunodeficiency virus, hepatitis B and C virus, and syphilis were negative. Chest X-ray was normal, and latent Mycobacterium tuberculosis infection was excluded at baseline with a negative interferon-gamma release assay. The second set of urine and blood cultures was negative (which included analysis for mycobacteria). Normal levels of serum angiotensin-converting enzyme excluded sarcoidosis. An autoimmunity profile, which included the presence of antinuclear antibodies, double-strand deoxyribonucleic acid antibodies, rheumatoid factor, cyclic citrullinated peptide antibodies, and evidence of complement deficiency, was also negative. 

**Figure 1:**
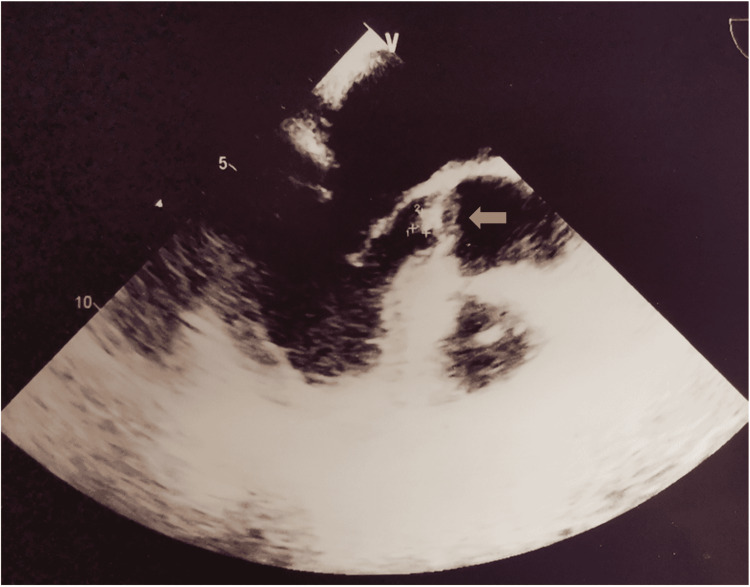
Transesophageal echocardiogram showing a tricuspid valve vegetation measuring 5.3 x 5.6 mm

A thyroid ultrasound identified a multinodular gland and a total body computed tomography scan (CT scan) was negative for suspicious lesions. A transthoracic echocardiogram identified vegetation on the tricuspid valve, confirmed with a transesophageal echocardiogram (Figure 1).

Infective endocarditis (IE) was assumed, and an empirical antibiotic regimen was started, including gentamycin, vancomycin, and ciprofloxacin. Due to persistent fever, the antibiotic therapy was changed to meropenem, doxycycline, and amphotericin B, while the previous diagnostic approach was revised. At this point, infection from fastidious organisms, including those of the HACEK group (Haemophilus species, Aggregatibacter species, Cardiobacterium hominis, Eikenella corrodens, and Kingella species) and fungal infection were considered and excluded with persistently negative blood cultures. Aspergillus galactomannan antigen test was negative.

**Figure 2:**
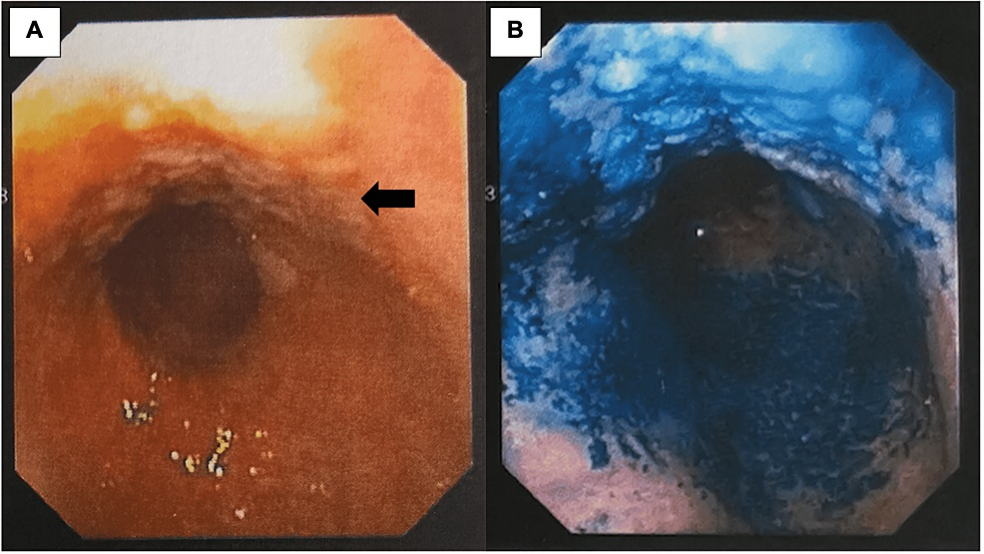
Upper digestive endoscopy of the gastric lesion Upper gastric endoscopy identified an antral lesion with a "lateral spreading" pattern and longitudinal spreading of 90 mm before (A) and after methylene blue instillation, with irregular capture at the lesion site (B).

An upper gastric endoscopy identified an extensive antral lesion suggestive of an infiltrative neoplastic disease, which was biopsied (Figure 2).

The histology identified a tubulovillous adenoma with low-grade dysplasia and focal high-grade dysplasia with metaplasia of the adjacent mucosa. Non-infectious thrombotic endocarditis (NTBE) was assumed in the probable context of gastric cancer, and the patient was proposed for a diagnostic laparoscopy. Peritoneal fluid was positive for reactive mesothelial cells and histocytes but negative for neoplastic cells. The patient was then proposed for sub-total gastrectomy, which was successfully performed. Histology identified a gastric mixed adenocarcinoma (OMS 2010 criteria) with a tubular-intestinal pattern (80%) and a diffuse component of signet ring cells (20%). Invasion of submucosa and one local metastasized lymph node was identified (pT1N1Mx). Later, the patient was submitted to a total gastrectomy with D2 lymphadenectomy (histology excluded residual disease). Resolution of initial symptoms was verified, including fever. 

During the first two months after the last surgery, the patient maintained anorexia with progressive weight loss. To ensure nutritional support, she was kept under parenteral nutrition before the surgery and enteral nutrition with a nasogastric tube after the surgery. Seven days after the procedure, new onset of fever was noted with signs of infection of the surgical wound. An abdominal CT scan identified a splenic abscess, treated with a conservative approach due to her frail condition. Clinical and laboratory improvement was noted, and after almost six months of hospitalization, the patient was discharged to a nursing home.

## Discussion

This is a case of NBTE secondary to an early-stage gastric carcinoma without other associated thrombotic phenomena. Gastric cancer remains a major health problem and a common cause of death and morbidity. It appears to progress slowly, and symptoms frequently manifest in the advanced stages of the disease [8]. The association between thrombotic phenomena and cancer is well established, and adenocarcinoma represents the most common histologic type of related neoplasm in NBTE patients [2]. A higher prevalence of IE, the presenting symptoms, and the first set of negative laboratory and imaging exams for autoimmune or neoplastic diseases may explain an initial presumptive diagnosis of IE and support the use of antibiotics. However, the persistence of fever, serial negative cultures, and a high level of suspicion for other non-infective conditions helped to define the diagnosis, which allowed for a timely referral and treatment. 

The antemortem diagnosis of NBTE is challenging, with 1,2% to 3,4% of cases identified in the autopsy series [2,7,9-11]. However, post-mortem pathology records suggest that NBTE may be more frequent than IE, raising concern about the diagnostic approach to patients with persistent fever syndromes [10,12]. It implies not only ruling out infection but also the identification of vegetations using echocardiography. In the case of NBTE, this can be difficult since vegetations tend to be more friable and often embolized compared with IE vegetations. For this reason, thromboembolic events are frequently the first manifestation of NBTE but can also reveal an advanced neoplastic disease [13]. In fact, a fatal event may be the first manifestation of endocarditis, independently of the etiology, which may explain the higher post-mortem diagnostic rate of NBTE [10].

From the standpoint of pathophysiology, NBTE and IE vegetations components appear to be similar, which suggests a probable common mechanism. Consequently, it is understandable that cancer or other inflammatory chronic diseases may cause NBTE but also predispose to IE [10]. In another way, cancer-related endocarditis can occur in the context of undiagnosed infective endocarditis due to the frequent exposure to prophylactic antibiotics in cancer patients or the presence of fastidious micro-organisms [14]. For this reason, it is difficult to estimate the true prevalence of cancer-related NBTE. Irrespective of etiology, without treatment, endocarditis may lead to valve dysfunction, systemic embolism, and, ultimately, death [15]. The pathogenesis of NBTE is not clearly defined, but it seems to involve immunologic mechanisms that potentiate the formation of thrombi which may proceed to form sterile vegetations on heart valves. There is no clear predisposition for a specific heart valve, but right-side valves have been less documented [12]. Furthermore, there is no firm correlation between previous valve disease and NBTE, which often occurs in otherwise healthy valves [10,16]. Associated systemic emboli phenomena have a wide variation, which may depend on the underlying disease [13]. As for cardiovascular involvement, NBTE is paucisymptomatic and clinical manifestations derive more frequently from systemic emboli affecting cerebral, coronary, renal, and mesenteric circulations [12]. Heart murmurs and severe valvular dysfunction are rare, which complicates the antemortem diagnosis of NBTE [4,7]. 

Transesophageal echocardiography allows accurate and early identification of NBTE and is considered the gold-standard diagnostic method for endocarditis due to its high sensitivity [13]. In any case, a high level of suspicion remains the first and most important step in the diagnostic approach. Since NBTE may occur in the context of neoplastic diseases, it is mandatory to include cancer screening when approaching a patient with a recurrent fever with or without other signs of infection. The therapy for NBTE relies on three cornerstones: treatment of the underlying disease, systemic anticoagulation, and rarely, surgical intervention for valve replacement, reserved for severe valvular disease [13]. However, as cancer-related NBTE is frequently associated with metastatic disease, treatment is often limited and ineffective.

In the present case, NBTE was the first thrombotic manifestation of gastric cancer. The diagnosis of early-stage gastric cancer allowed for a timely referral and surgical treatment of the underlying disease. Despite the evidence of early-stage gastric cancer, the standard approach should include the use of neoadjuvant chemotherapy, independent of the tumor stage [17]. A very fragile general condition, the absence of metastatic disease and patient preference may have justified this approach by the surgical and oncology team. The patient remains on follow-up with no evidence of relapse. 

## Conclusions

The diagnosis of NBTE in a patient with persistent fever should prompt a search for other non-infective conditions, including malignancy. There are few reported cases of NBTE as a presenting manifestation of gastric cancer, and the majority occur in advanced disease. Our case illustrates a rare presentation of early-stage gastric cancer and highlights the importance of keeping a broad differential diagnosis and persistence in what concerns diagnostic algorithms. The antemortem diagnosis of NBTE allowed for a timely referral and treatment of the underlying disease.

## References

[1] Sánchez Quirós B, Ruiz López N, López Herrero R, Bartolomé Bartolomé C (2020). Marantic endocarditis. Rev Esp Anestesiol Reanim.

[2] Deppisch LM, Fayemi AO (1976). Non-bacterial thrombotic endocarditis: clinicopathologic correlations. Am Heart J.

[3] Thomas RH (2001). Hypercoagulability syndromes. Arch Intern Med.

[4] Mazokopakis EE, Syros PK, Starakis IK (2010). Nonbacterial thrombotic endocarditis (marantic endocarditis) in cancer patients. Cardiovasc Hematol Disord Drug Targets.

[5] Edoute Y, Haim N, Rinkevich D, Brenner B, Reisner SA (1997). Cardiac valvular vegetations in cancer patients: a prospective echocardiographic study of 200 patients. Am J Med.

[6] Yusuf SW, Ali SS, Swafford J (2006). Culture-positive and culture-negative endocarditis in patients with cancer: a retrospective observational study, 1994-2004. Medicine.

[7] Le Bot A, Jégo P, Donal E, Flécher E, Revest M, Tattevin P (2018). Non-infective endocarditis. Rev Med Interne.

[8] Rawla P, Barsouk A (2019). Epidemiology of gastric cancer: global trends, risk factors and prevention. Prz Gastroenterol.

[9] Smeglin A, Ansari M, Skali H, Oo TH, Maysky M (2008). Marantic endocarditis and disseminated intravascular coagulation with systemic emboli in presentation of pancreatic cancer. J Clin Oncol.

[10] Bussani R, DE-Giorgio F, Pesel G (2019). Overview and comparison of infectious endocarditis and non-infectious endocarditis: a review of 814 autoptic cases. In Vivo.

[11] Fournier PE, Thuny F, Richet H (2010). Comprehensive diagnostic strategy for blood culture-negative endocarditis: a prospective study of 819 new cases. Clin Infect Dis.

[12] el-Shami K, Griffiths E, Streiff M (2007). Nonbacterial thrombotic endocarditis in cancer patients: pathogenesis, diagnosis, and treatment. Oncologist.

[13] Lopez JA, Ross RS, Fishbein MC, Siegel RJ (1987). Nonbacterial thrombotic endocarditis: a review. Am Heart J.

[14] Werner M, Andersson R, Olaison L, Hogevik H (2003). A clinical study of culture-negative endocarditis. Medicine.

[15] Hurrell H, Roberts-Thomson R, Prendergast BD (2020). Non-infective endocarditis. Heart.

[16] Patel MJ, Elzweig J (2020). Non-bacterial thrombotic endocarditis: a rare presentation and literature review. BMJ Case Rep.

[17] Cunningham D, Allum WH, Stenning SP (2006). Perioperative chemotherapy versus surgery alone for resectable gastroesophageal cancer. N Engl J Med.

